# Two New Indole Alkaloids from Toad Venom of *Bufo bufo gargarizans*

**DOI:** 10.3390/molecules25194511

**Published:** 2020-10-01

**Authors:** Yu-Lin Chen, Ying-Hui Dai, An-Dong Wang, Zi-Ying Zhou, Miao Lei, Jiao Liu, Bin Lin, Ming-Yu Xia, Dong Wang

**Affiliations:** 1 Faculty of Traditional Chinese Medicine, Shenyang Pharmaceutical University, Benxi 117004, China; cyl855@163.com (Y.-L.C.); yhdai2008@aliyun.com (Y.-H.D.); wangandong19891220@163.com (A.-D.W.); zzyspu@foxmail.com (Z.-Y.Z.); lemia1517@163.com (M.L.); jiao-l@foxmail.com (J.L.); 2 Faculty of Pharmaceutical Engineering, Shenyang Pharmaceutical University, Benxi 117004, China; randybinlin@sina.com; 3 Faculty of Life Sciences and Biological Pharmacy, Shenyang Pharmaceutical University, Shenyang 110016, China

**Keywords:** *Bufo bufo gargarizans*, toad venom, Bufotenidine B, Bufocarboline A, cytotoxic activity

## Abstract

Two new indole alkaloids, Bufotenidine B (**2**) and Bufocarboline A (**6**), along with seven known indole alkaloids (**1**, **3–5**, and **7–9**) and three organic acids (**10–12**), were isolated from the water extract of toad venom. The structures of the new alkaloids were elucidated by extensive spectroscopic methods. The absolute configurations of **4**, **6**, and **8** were determined for the first time by electronic circular dichroism (ECD) calculations. The cytotoxic activity of all compounds was tested against human malignant melanoma cells A375 by the MTT method, and no antitumor activity was observed.

## 1. Introduction

Toad venom (ChanSu in Chinese) is secreted by the skin or parotid glands of giant toads, such as *Bufo bufo gargarizans* Cantor or *B. melanostrictus* Schneider [1], and it is used by toads as chemical weapons against predators. It has been used as traditional Chinese medicine to treat tumors, carbuncles, scrofula, and heart failure [2]. Toad venom has been reported to contain various chemical constituents, including sterols, bufadienolides, indole alkaloids, and organic acids. Among them, bufadienolides are a type of liposoluble components, which are a class of polyhydroxy steroids with an α-pyrone ring at the C17 position and are considered to be its primary bioactive constituents with remarkable anti-tumor effect [3,4]. A water-soluble preparation of toad venom, ChanSu injection, which contains only trace amounts of bufadienolides, is used as an effective antitumor agent in clinics [5]. This suggests that there might be other components besides bufadienolides that exert cytotoxic activity in the water extract of toad venom. The alkaloids isolated from toad venom are all almost derivatives of serotonin with high hydrophilicity, in which bufoserotonin C exhibited cytotoxic effects against human lung adenocarcinoma epithelial cells A549 [6]. Further search for water-soluble antitumor components will be an important work for toad venom research

**Figure 1:**
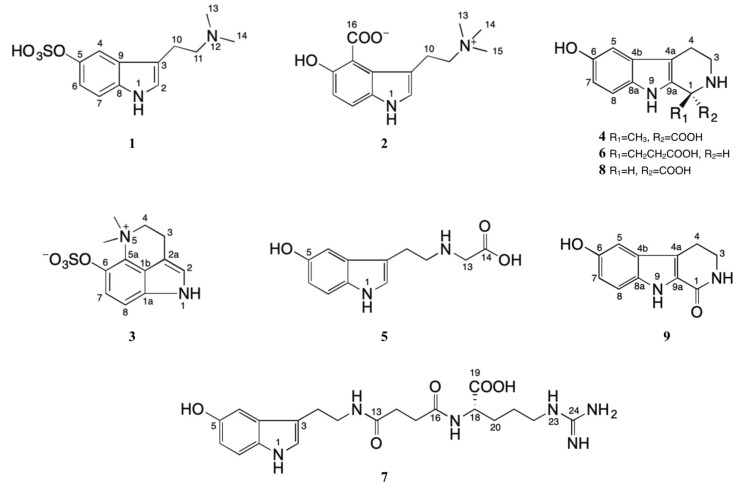
Chemical structures of compounds **1–9.**

In this study, two new indole alkaloids (**2** and **6**) along with seven known indole alkaloids (**1**, **3–5**, and **7–9**) and three organic acids (**10–12**), were isolated from the water extract of toad venom (Figure 1). The absolute configuration of three tetrahydro-β-carbolines was determined for the first time by electronic circular dichroism (ECD) calculations, and the cytotoxicity of these compounds against human malignant melanoma cells A375 was assayed.

## 2. Results and Discussion

**Figure 2:**
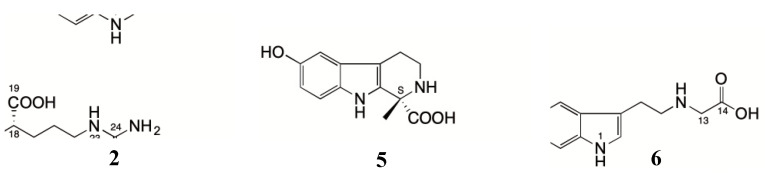
Key HMBC correlations of compounds **2**, **5** and **6.**

Compound **2** was afforded as a yellow powder. The molecular formula C_14_H_18_N_2_O_3_ was established by HR-ESI-MS spectrometry at *m/z* 263.1389[M+H]^+^ (C_14_H_19_N_2_O_3_, calculated 263.1390), 285.1208 [M+Na]^+^ (C_14_H_18_N_2_NaO_3_, calculated 285.1209) and 547.2526 [2M+Na]^+^ (C_28_H_36_N_4_NaO_6_, calculated 547.2527). The IR spectrum showed absorptions of NH group (3430 cm^−1^), carbonyl groups (1631 cm^−1^), and aromatic ring (1458, 1117, 1038 cm^−1^). The UV spectrum showed maximal absorption wavelength at 204, 232.5 and 312.4 nm. The ^1^H-NMR [DMSO-*d*_6_: D_2_O (1:4), 600 MHz] spectrum showed three aromatic proton signals at *δ*_H_7.31 (d, *J* = 8.7 Hz, 1H, H-7), 7.15(s, 1H, H-2), and 6.63 (d, *J* = 8.1 Hz, 1H, H-6), combining with eight carbon signals at *δ*_C_ 154.8 (C-5), 133.7 (C-8), 128.9 (C-2), 125.4 (C-9), 118.1 (C-7), 113.6 (C-6), 112.9 (C-4), and 110.7(C-3) in ^13^C-NMR [DMSO-*d*_6_:D_2_O (1:4), 150 MHz] spectrum, which indicated there was an indole moiety in **2**. The DEPT (135°) displayed three aromatic methine carbon signals at *δ*_C_ 128.9 (C-2), 118.1 (C-7), and 113.6 (C-6), and HSQC showed they correlated with 7.15 (s, 1H, H-2), 7.31 (d, *J* = 8.7 Hz, 1H, H-7), and 6.63 (d, *J* = 8.1 Hz, 1H, H-6), respectively. Combining with the HMBC correlations between 7.31 (d, *J* = 8.7 Hz, 1H, H-7) and 125.4 (C-9) and 154.8 (C-5); between 7.15(s, 1H, H-2) and 133.7 (C-8), 125.4 (C-9) and 110.7(C-3); and between 6.63 (d, *J* = 8.1 Hz, 1H, H-6) and 118.1 (C-7) and 112.9 (C-4), confirmed there was a 3, 4, 5- trisubstituted indole moiety in **2**. The DEPT (135°) spectrum disclosed the presence of a pair of coupled methylene groups at *δ*_C_ 23.6 (C-10) and 69.2 (C-11), which correlated with 3.32 (m, 2H, H-10), 3.23 (m, 2H, H-11) in its HSQC spectrum. Combining with the HMBC correlations between 3.32 (m, 2H, H-10) and 69.2 (C-11), and between 3.23 (m, 2H, H-11) and 23.6 (C-10) indicated the two methylene groups were directly connected. In addition, the HMBC correlations between 7.15 (s, 1H, H-2) and 23.6 (C-10); between 3.32 (m, 2H, H-10) and 125.4 (C-9), 110.7(C-3), and 128.9 (C-2); and between 3.23 (m, 2H, H-11) and 110.7 (C-3) disclosed a pair of coupled methylene groups was attached to the 3, 4, 5- trisubstituted indole moiety at C-3, thus forming a 5-hydroxytryptamine moiety. The HSQC spectrum showed 2.98 (s, 16H, N-CH_3_) correlated with 54.6 (C-13, 14, and 15). Meanwhile, the DEPT (135°) displayed 54.6 (C-13, 14, and 15) was a positive peak, and the peak height about three times higher than that of other carbon signals, suggesting that there were three identical methyl groups in **2**. Finally, three identical methyl groups were connected to the 5-hydroxytryptamine moiety at N-2 by the HMBC correlations between 3.23 (m, 2H, H-11) and 54.6 (C-13, 14, 15), and between 2.98 (s, 16H, N-CH_3_) and 69.2 (C-11) (Figure 2).

According to the aforementioned information, a structural fragment of C_13_H_18_N_2_O which missed a [COO^−^] fragment compared to the molecular formula C_14_H_18_N_2_O_3_ of **2** was formed. The ^13^C-NMR spectrum showed the presence of a carbonyl group at *δ*_C_ 176.9, suggesting the [COO^-^] fragment was attached to C-4 to form an inner salt. The suggestion was further demonstrated by its UV spectrum, which showed that the maximum absorption wavelength of the indole ring shifted from 275 nm to 312 nm. This indicated that the C-4 substituent could prolong the conjugated system. Based on the above analysis, **2** was unambiguously assigned as 5-hydroxy-3-(2-(trimethylammonio) ethyl) -1H-indole-4-carboxylate, and named bufotenidine B, which is a new compound confirmed by Scifinder investigation.

Compound **5** was obtained as a brown powder. The molecular formula was established as C_12_H_14_N_2_O_3_ by high-resolution electrospray ionization mass spectroscopy, which gave ions at *m/z* 467.19261 [2M-H]^−^ (C_24_H_27_N_4_O_6_, calculated 467.19361). The IR bands of **5** were shown at 3424, 1631 and 1457 cm^−1^ for N-H, C=O and aromatic ring, respectively. The UV spectrum showed maximal absorptions of an indole chromophore (222 and 276 nm). In the ^1^H-NMR [DMSO-*d*_6_:D_2_O (10:3), 600 MHz, *δ*_H_] spectrum of **5**, signals at 7.15 (d, *J* = 8.6 Hz, 1H, H-7), 7.08 (s, 1H, H-2), 6.84 (d, *J* = 2.1 Hz, 1H, H-4), and 6.60 (dd, *J* = 8.6, 2.2 Hz,1H,H-6) indicated a typical 3,5-disubstituted indole moiety. Combined with two methylene signals at 3.07 (t, *J* = 7.2 Hz, 2H, H-11) and 2.91 (t, *J* = 7.2 Hz, 2H, H-10), it was suggested that **5** is a derivative of serotonin. The ^13^C-NMR [DMSO-*d*_6_:D_2_O (10:3), 150 MHz, *δ*_C_] spectrum showed twelve carbon signals. Ten of them at 124.6 (C-2), 109.3 (C-3), 103.0 (C-4), 150.8 (C-5), 112.5 (C-6), 113.0 (C-7), 131.7 (C-8), 128.3 (C-9), 22.6 (C-10), and 47.8 (C-11) were assigned to serotonin skeleton by comparing with the NMR data of serotonin [7]. The ^1^H-NMR showed one methylene signal at *δ*_H_ 3.30 (s, 2H, H-13) which corresponded to 50.1 (C-13) in HSQC spectrum. Combining with the HMBC correlations between 3.30 (s, 2H, H-13) and 169.1 (C-14), disclosed the presence of one carboxymethyl group, which was attached to the serotonin skeleton at N-2 due to the HMBC correlations between 3.07 (t, *J* = 7.2Hz, 2H, H-11) and 50.1 (C-13), and between 3.30 (s, 2H, H-13) and 47.8 (C-11) (Figure 2). Therefore, **5** was clearly assigned to 2-(5-hydroxy-1H-indol-3-yl)ethyl)glycine, named *N*-carboxymethyl serotonin. It was a new natural product.

Compound **6** was obtained as a pale white powder. The molecular formula was determined as C_14_H_16_N_2_O_3_ on the basis of the presence of a quasi-molecular ion at *m/z* 261.1232 [M+H]^+^ (C_14_H_17_N_2_O_3_, calculated 261.1234), 259.1078 [M-H]^−^ (C_14_H_15_N_2_O_3_, calculated 259.1088) in its HR-ESI-MS spectrum. Characteristic IR absorption bands indicated the existence of aromatic secondary amine (3404 cm^−1^), carbonyl (1631 cm^−1^) and aromatic ring (1457 cm^−1^) groups. The UV spectrum exhibited maximal absorptions of an indole chromophore (221 and 275 nm). The ^13^C-NMR (DMSO-*d*_6_, 150 MHz) showed fourteen carbon signals for one carbonyl group at *δ*_C_ 175.3 (C-12), five sp3 carbons at *δ*_C_ 51.9 (C-1), 39.2 (C-3), 20.4 (C-4), 28.2 (C-10), 33.9 (C-11), and eight sp2 carbons at *δ*_C_ 134.1 (C-9a), 106.4 (C-4a), 127.3 (C-4b), 102.2 (C-5), 150.6 (C-6), 111.2 (C-7), 111.5 (C-8), and 130.5(C-8a). The ^1^H-NMR (DMSO-*d*_6_, 600 MHz) spectrum indicated the presence of one NH group as a singlet [*δ*_H_10.54 (1H, s, H-9)], and three aromatic protons [7.07 (d, *J* = 8.6 Hz, 1H, H-8), 6.69 (d, *J* = 2.0 Hz, 1H, H-5), 6.55 (dd, *J* = 8.5, 2.2Hz, 1H, H-7)], disclosed a 2,3,5-trisubstituted indole moiety, which was supported by the HMBC correlations between 10.54 (1H, s, H-9) and 134.1 (C-9a), 130.5(C-8a), 127.3 (C-4b), and 106.4 (C-4a); between 6.69 (d, *J* = 2.0 Hz, 1H, H-5) and 106.4 (C-4a), 150.5(C-6), 111.2 (C-7), and 130.5(C-8a); between 6.55 (dd, *J* = 8.5, 2.2Hz, 1H, H-7) and 130.5(C-8a), 150.5(C-6), and 102.2 (C-5); and between 7.07 (d, *J* = 8.6 Hz, 1H, H-8) and 102.2 (C-5), 127.3 (C-4b), and 150.6 (C-6). ^1^H-NMR spectrum indicated two methylene signals at *δ*_H_ 2.67–2.54 (m, 2H, H-4), 3.20–2.96 (m, 2H, H-3), which, respectively, correlated with 20.4 (C-4), 39.2(C-3) in HSQC spectrum. Combining the HMBC correlations between 2.67–2.54 (m, 2H, H-4) and 39.2 (C-3), 134,1 (C-9a), and 106.4 (C-4a) and between 3.20–2.96 (m, 2H, H-3) and 20.4 (C-4) and 106.4 (C-4a), it disclosed a pair of coupled methylene groups was attached to the 2, 3, 5-trisubstituted indole moiety at C-3, thus forming a 5-hydroxytryptamine moiety. ^1^H-NMR spectrum indicated two methylene signals at *δ*_H_ 2.40–2.21 (m, 2H, H-11), 1.90–2.10 (m, 2H, H-10), which respectively correlated with 33.9 (C-11) and 28.2 (C-10) in HSQC spectrum. The HMBC gave correlations between 2.40–2.21 (m, 2H, H-11) and 28.2 (C-10) and 175.3 (C-12) and between 1.90–2.10 (m, 2H, H-10) and 33.9 (C-11) and 175.3 (C-12), which disclosed the presence of a carboxyethyl group. ^1^H-NMR spectrum showed a methine signal at *δ*_H_ 4.19 (d, *J* = 8.0Hz, 1H, H-1), which correspond to 51.9 (C-1) in HSQC spectrum. In addition, the HMBC spectrum showed two methylene groups 2.40–2.21 (m, 2H, H-11) and 1.90–2.10 (m, 2H, H-10) were both associated with 51.9 (C-1), indicating that the carboxyethyl is connect to C-1 (51.9). Further, the HMBC spectrum showed correlations between 4.19 (d, *J* = 8.0Hz, 1H, H-1), 1.90-2.10 (m, 2H, H-10) and 134.1 (C-9a), showing that 51.9 (C-1) is linked to 134.1 (C-9a) to form a tricyclic structure of β-carboline (Figure 2). According to the aforementioned information, the plane structure of **6** was unambiguously assigned as 6-hydroxy-1-(2′-carboxyethyl)-1,2,3,4-tetrahydro-β-carboline, which is a new compound named bufocarboline A.

**Figure 3:**
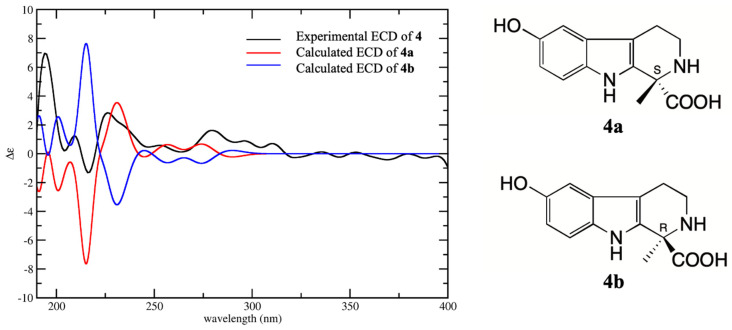
Experimental CD spectrum of **4** in H_2_O and the calculated ECD spectra of (1*S*)-4 (**4a**) and (1*R*)-4 (**4b**).

**Figure 4:**
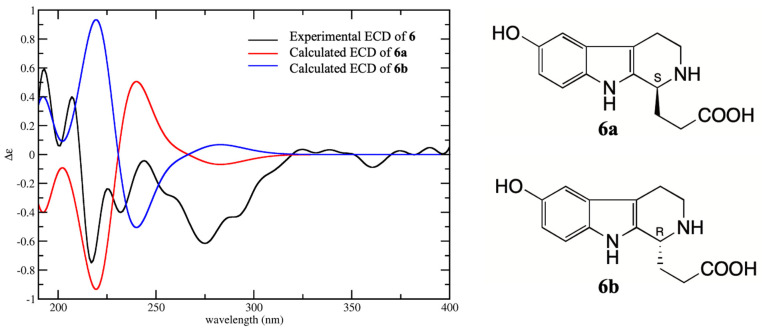
Experimental CD spectrum of **6** in H_2_O and the calculated ECD spectra of (1*S*)-6 (**6a**) and (1*R*)-6 (**6b**).

**Figure 5:**
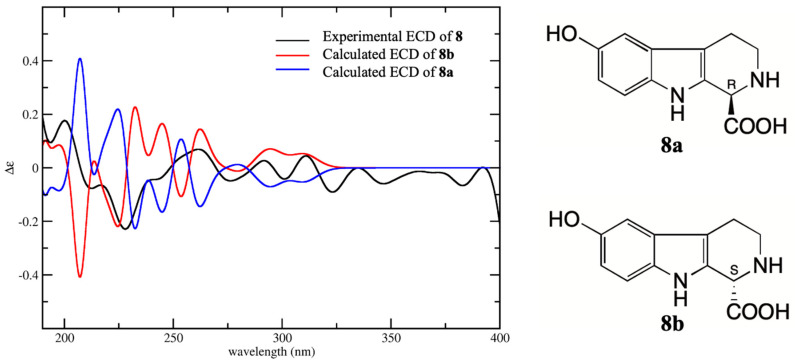
Experimental CD spectrum of **8** in H_2_O and the calculated ECD spectra of (1*R*)-8 (**8a**) and (1*S*)-8 (**8b**).

Compounds **4** and **8** were isolated for the first time from toad venom, and the plane structures of them were confirmed respectively as 6-hydroxy-l-methyltetrahydro-β-carboline-1-carboxylic acid [8] and 6-Hydroxy-2,3,4,9-tetrahydro-1H-β-carboline-1-carboxylic acid [8] by the comparison with extensive spectroscopic data. The chemical synthesis and biological activities of them have been reported in the literature [9,10,11], but the absolute configurations have not been determined. Compounds **4**, **6**, and **8** are all derivatives of tetrahydro-β-carboline with one chiral carbon at C-1. The absolute configurations of **4**, **6**, and **8** were determined by comparison with their experimental CD spectra and calculated electronic circular dichroism (ECD) spectra, which were calculated by a quantum chemical method. Compound **4** has two possible enantiomers (1*S*)-4 (**4a**) and (1*R*)-4 (**4b**), and the calculated ECD curve of **4a** was similar to the experimental CD spectrum, both showing Cotton effects at 205–220 (negative) and 220–240 nm (positive) (Figure 3). Therefore, the absolute configuration **4** was assigned as 1*S*. The structure of **6** was similar to **4** except for the loss of methyl at C-1 instead of carboxyethyl. The calculated ECD spectrum of enantiomer (1*S*)-6 (**6a**) was in good agreement with the experimental spectrum, both showing negative Cotton effect at 210–230 and 260–300 nm and positive Cotton effect 230–260 nm (Figure 4). Therefore, the absolute configuration of **6** was assigned as 1*S*. Compound **8** was a demethylated derivative of **4**. Considering the biosynthesis of **4** and **8** in *B. bufo gargarizans*, it could be concluded that the stereoconfiguration of carboxyl group at C-1 of **8** should be the same as that of **4**. The comparison of the CD and ECD spectra of enantiomers (1*R*)-8 (**8a**) and (1*S*)-8 (**8b**) showed that the calculated ECD spectra of **8b** was matched better with that of CD than **8a** (Figure 5). Furthermore, the calculated specific rotation value of **8a** was 103.7, and the calculated specific rotation value of **8b** was corresponded to −103.7, which yielded a good match with the measured value ${\left[\alpha \right]}_{D}^{20}$−115.2 (c, 0.033, H_2_O) of **8.** Consequently, the absolute configuration of **8** was assigned as 1*S*. The absolute configurations of **4**, **6**, and **8** were all identified as 1S, indicating they have similar biosynthesis pathways.

Compound **1** was obtained as a yellow powder. The ^1^H-NMR [DMSO-*d*_6_, 600 MHz, *δ*_H_] spectrum indicated the presence of one NH group as a singlet at 10.83 (s, 1H, H-1), and four aromatic protons at 7.36 (d, *J* = 2.1 Hz, 1H, H-2), 7.22 (d, *J* = 8.7 Hz, 1H, H-7), 7.20 (d, *J* = 2.2 Hz, 1H, H-4), and 6.97 (dd, *J* = 8.7 Hz, 2.2 Hz,1H, H-6)], indicated a typical 3,5-disubstituted indole moiety. Combined with two methylene signals at 3.27 (t, *J* = 7.2 Hz, 2H, H-11) and 3.00 (t, *J* = 7.2 Hz, 2H, H-10), it was suggested that **1** is a derivative of serotonin. In addition, there was two methyl signals at 2.82 (s, 6H, H-13, H-14)]. The ^13^C-NMR [DMSO-*d*_6_, 150 MHz, *δ*_C_] spectrum showed twelve carbon signals at 20.6 (C-10), 57.1 (C-11), 109.0 (C-4), 110.3 (C-3), 111.2 (C-6), 116.9 (C-7), 124.1 (C-2), 126.8 (C-9), 133.4 (C-8), 146.6 (C-5), 42.6 (C-13, C-14) were basically consistent with bufotenine [10]. However, the ESI-MS showed its [M+H]^+^, [M+Na]^+^ and [M-H]^-^ located at *m/z* 284.9, 306.9 and 282.6 in positive or negative mode, respectively, which confirmed the molecular weight of **1** was 284 instead of 204 (the molecular weight of bufotenine). Thus, **1** was identified as bufotenine O-sulfate known as bufoviridine. This is the first time to report the NMR data about bufoviridine.

The other six compounds **3**, **7**, **9–12** were identified as dehydrobufothionine [12], bufobutarginine [6], 6-Hydroxy-1-oxo-3,4-dihydro-β-carboline [13], 1,7-pimelic acid [14], suberic acid [15], azelaic acid [16] in comparison with their NMR data in the literature respectively.

The cytotoxic activities of **1–12** were evaluated against human malignant melanoma cells A375 using the MTT method. Unfortunately, the experimental results disclosed that the IC_50_ values of them were greater than 100 μM, and no antitumor activity was observed. In our previous study, we isolated one organic acid and ten bufotenines from the water extract of the toad venom. Among them, only bufoserotonin C exhibited cytotoxic effects against human lung adenocarcinoma epithelial cells A549 with IC_50_ of 34.3 µM than that of positive control 5-FU (IC_50_ of 48.65 μM) [12]. There we speculated that the antitumor activity of water extract and water-soluble preparations of toad venom may be manifested by the trace bufadienolides which possessed powerful cytotoxicity despite their solubility being very low in water.

## 3. Materials and Methods

### 3.1. General Information

Optical rotation was measured on an Anton Paar MCP200 Polarimeter (Anton Paar Co., Austria). UV spectra were recorded on a Shimadzu UV-1700 Spectrophotometer (Shimadzu Co., Kyoto, Japan). IR spectra were recorded on a Bruker IFS 55 FTIR spectrometer on KBr pellets (Bruker Co., Karlsruhe, Germany). CD spectrum was recorded by a MOS 450 detector (Bio-Logic Co., Claix, France). NMR spectra were recorded on Bruker ARX-600 spectrometer (chemical shift values are presented as δ values with TMS as the internal standard; Bruker Co., Billerica, MA, USA). HR-ESI-MS data were recorded on a Waters Xevo G2 Q-TOF mass spectrometer (Waters Co., Milford, MA, USA). Semi-preparative HPLC was performed on a Model LC-10ATVP system consisting of two LC-10AT HPLC pumps with a SPD-10Avp detector. Phenomenex Luna C18 column (250 × 10.00 mm, 5 μm), Venusil HILIC column (10 μm, 100 Å, 10 × 250 mm) and Kinetex^®^ 5μm Biphenyl column (100 Å, 250 × 10.0 mm) were used for preparation. Column chromatography was performed using 101 macroporous resin (0.3–1.25 mm, Cangzhou Bon Adsorber Technology Co., Ltd, Cangzhou, China), Sephadex LH-20 (40–70 μm, Amersham Pharmacia Biotech AB, Uppsala, Sweden). TLC was conducted on silica gel GF 254 (Marine Chemical Factory, Qingdao, China) plates. All solvents used in column chromatography and HPLC were of analytical grade (Tianjin Yongda Chemical Reagent Co., Ltd, Tianjin, P. R. China) and chromatographic grade (Tianjin Concord Technology Co., Ltd, Tianjin, P. R. China), respectively.

### 3.2. Materials

The crude drug ChanSu was collected from Linyi, Shandong Province, China, in March 2010 and authenticated by the Associate Professor Dong Wang from Shenyang Pharmaceutical University. Human malignant melanoma cells A375 were purchased from the American Type Culture Collection (ATCC, Rockville, MD, USA).

### 3.3. Extraction and Isolation

The dried and roughly powdered ChanSu (250 g) was extracted with dichloromethane (7 × 2.5 L) under reflux. The solvent was removed in vacuo to afford dichloromethane extract (50 g). The residue (200 g) was extracted 6 times by distilled water (6 × 2 L) with an ultrasonator (200 W, 59 kHz, 30 min), and concentrated in vacuo to obtain crude water extract (120 g). The crude water extract was suspended with 1 L distilled water and partitioned 3 times with 1 L of *n*-butanol saturated with water. Collected water phase and concentrated under vacuum to afford water extract (100 g). The water extract was dispersed with 0.5 L distilled water, and added ethanol to a final concentration of 75% (*v*/*v*), then kept for 12 h at 4 °C. The filtrate was evaporated to give water soluble low molecular components (WSLM) (50 g). The WSLM was subjected to 101 macroporous resin, adsorbed overnight, and then eluted with water–ethanol (100:0, 70:30, 40:60, and 5:95, *v*/*v*) successively to yield 4 fractions, Fr. 1 (35.0 g), Fr. 2 (9.4 g), Fr. 3 (4.6 g), and Fr. 4 (1.0 g). Fr. 2 (9.4 g) was dispersed with 100 mL distilled water, and partitioned 3 times with 100 mL ethyl acetate. The water phase and ethyl acetate were collected and concentrated under vacuum to afford the water extract (8.7 g) and the ethyl acetate extract (670 mg). 

The ethyl acetate extract (670 mg) was further separated by semi-preparative HPLC (MeOH-H_2_O, 40:60, Biphenyl column, 1 mL/min, 230 nm) to obtain **11** (5 mg, *t*_R_ 16 min). The water extract (8.7 g) applied to Sephadex LH-20 (methanol-water, 40:60, *v*/*v*) to get twenty-four sub-fractions (sub-Fr. 2-1-24). The sub-Fr. 2-6 (278.4 mg) was further separated by semi-preparative HPLC (MeOH-H_2_O, 10:90, Biphenyl column, 3 mL/min, 230 nm) and purified by semi-preparative HPLC (MeCN-H_2_O, 88:12, Hilic column, 3 mL/min, 230 nm) to yield **1** (6.7 mg, *t*_R_ 13 min) and **2** (1.2 mg, *t*_R_ 14.5 min). The sub-Fr. 2-7 (132 mg) was further purified by semi-preparative HPLC (MeOH-H_2_O, 8:92, ODS column, 3 mL/min, 230 nm) to obtain **3** (10 mg, *t*_R_ 28.6 min). The sub-Fr. 2-8 (152.1 mg) was subjected to semi-preparative HPLC with a gradient elution of MeOH-H_2_O (7:93, 0–55 min; 17:83, 56–80 min, ODS column, 3 mL/min, 230 nm) to afford six sub-sub-fractions (sub-sub-Fr. 2-8-1-6). The sub-sub-Fr. 2-8-2 (25 mg) was further separated by semi-preparative HPLC (MeOH-H_2_O, 10:90, Biphenyl column, 3 mL/min, 230 nm) to yield **4** (10 mg, *t*_R_ 12.6 min) and **5** (14 mg, *t*_R_ 16.1 min). The sub-sub-Fr. 2-8-4 (20 mg) was purified by semi-preparative HPLC (MeCN-H_2_O, 70:30, Hilic column, 3 mL/min, 230 nm) to give **6** (8 mg, *t*_R_ 7.5 min). The sub-sub-fr.2-8-6 (21.3 mg) was purified by semi-preparative HPLC (MeCN-H_2_O, 80:20, Hilic column, 3 mL/min, 230 nm) to afford **7** (14 mg, *t*_R_ 14 min). The sub-Fr. 2-9 (53.8 mg) was further separated by semi-preparative HPLC (MeOH-H_2_O, 5:95, Biphenyl column, 3 mL/min, 230 nm) and purified by analytical HPLC (MeOH-H_2_O, 5:95, Phenyl-hexyl column, 1 mL/min, 230 nm) to yield **8** (1 mg, *t*_R_ 21.7 min). The sub-Fr. 2-16 (48.6 mg) was further purified by semi-preparative HPLC (MeCN-H_2_O, 95:5, Hilic column, 3 mL/min, 230 nm) to obtain **9** (1.3 mg, *t*_R_ 10 min) and **12** (2 mg, *t*_R_ 8 min). The sub-Fr. 2-23 (35 mg) was further separated by semi-preparative HPLC (MeCN-H_2_O, 98:2, Hilic column, 3 mL/min, 230 nm) to obtain **10** (3 mg, *t*_R_ 11 min).

#### 3.3.1. Bufoviridine (**1**)

Yellow powder; ESI-MS *m*/*z* 284.9[M+H]^+^, 306.9[M+Na]^+^ and 282.6[M-H]^−^, C_12_H_16_N_2_O_4_S. ^1^H-NMR and ^13^C-NMR spectral data as shown in Table 1.

#### 3.3.2. Bufotenidine B (**2**)

Yellow powder; HR-ESI-MS *m*/*z* 263.1389[M+H]^+^ (C_14_H_19_N_2_O_3_, calculated 263.1390), 285.1208 [M+Na]^+^ (C_14_H_18_N_2_NaO_3_, calculated 285.1209), 547.2526 [2M+Na]^+^ (C_28_H_36_N_4_NaO_6_, calculated 547.2527), C_14_H_18_N_2_O_3_; IR (KBr) ν_max_: 3430, 2924, 2170, 1698, 1631,1458, 1402, 1384, 1338, 1272, 1117, 1038, 865, 834, 669, 618 cm^−1^; UV (H_2_O) λ max (log ε): 204 (4.01), 232.5 (3.68), 312.4 (3.41) nm. ^1^H-NMR and ^13^C-NMR spectral data as shown in Table 1.

#### 3.3.3. *S*-6-hydroxy-l-methyltetrahydro-β-carboline-1-carboxylic Acid (**4**)

Yellow powder; ESI-MS *m*/*z* 244.6 [M-H]^-^, C_13_H_14_N_2_O_3_; ${\left[\alpha \right]}_{D}^{20}$-120 (c, 0.02, H_2_O). ^1^H-NMR and ^13^C-NMR spectral data are shown in Table 2.

#### 3.3.4. *N*-carboxymethyl Serotoin (**5**)

Yellow powder; ESI-MS *m*/*z* 234.9[M+H]^+^, 256.9 [M+Na]^+^, 232.6 [M-H]^−^, HR-MS *m*/*z* 467.19261[2M-H]^−^ (C_24_H_27_N_4_O_6_, calculated 467.19361), C_12_H_14_N_2_O_3_; IR (KBr) ν_max_: 3424, 2925, 2170, 2852, 1697, 1631, 1457, 1402, 1384, 1272, 1197, 1143, 1039, 865, 834, 704, 668, 618 cm^−1^; UV (H_2_O) λ max (log ε): 200.5 (4.21), 221.6 (4.06), 275.7 (3.51) nm. ^1^H-NMR, ^13^C-NMR, and HMBC spectral data are shown in Table 1.

#### 3.3.5. Bufocarboline A (**6**)

Yellow powder; HR-MS *m*/*z* 261.1232[M+H]^+^ (C_14_H_17_N_2_O_3_, calculated 261.1234), 283.1053 [M+Na]^+^, 259.1078 [M-H]^-^ (C_14_H_15_N_2_O_3_, calculated 259.1088), C_14_H_16_N_2_O_3_; IR (KBr) ν_max_: 3404, 2922, 2852, 2170, 1631, 1572, 1457, 1402, 1385, 1274, 1230, 1198, 1149, 1049, 864, 831, 798, 668, 621, 475 cm^−1^; UV (H_2_O) λ max (log ε): 204 (4.22), 274.5 (3.52), 221 (3.99) nm. ${\left[\alpha \right]}_{D}^{20}$-72.5 (c, 0.04, H_2_O). ^1^H-NMR and ^13^C-NMR spectral data are shown in Table 2.

#### 3.3.6. *S*-6-Hydroxy-2,3,4,9-tetrahydro-1*H*-β-carboline-1-carboxylic Acid (**8**)

Yellow powder; HR-MS *m*/*z* 233.0920 [M+H]^+^ (C_12_H_13_N_2_O_3_, calculated 233.0921), C_12_H_12_N_2_O_3_; ${\left[\alpha \right]}_{D}^{20}$-115.2 (c, 0.033, H_2_O); UV (H_2_O) λ max (log ε): 215.7 (3.52), 274.5 (2.93) nm. ^1^H-NMR and ^13^C-NMR spectral data are shown in Table 2.

### 3.4. ECD Calculations

For the computational details for ECD spectra of compounds **4**, **6**, and **8** based on their known relative configuration, all six possible stereoisomers were employed for the conformational random search using the MMFF94s force field by CONFLEX software package [17]. The conformational search results with an energy cut off of 3 kcal/mol were selected to further geometry optimization and ECD calculation. The initial conformers were optimized at B3LYP/6-31G(d) theoretical level by using Gaussian09 software [18]. They were then checked by frequency calculation and resulted in no imaginary frequencies. The ECD of the conformers were then calculated by the TDDFT method at the B3LYP/6-311++G(2d,p) level with the CPCM model in methanol solution. The calculated ECD curve was generated based on Boltzmann weighted average of the conformations search results using SpecDis 1.51 [19].

## Figures and Tables

**Table 1:** ^1^H and ^13^C-NMR data (600/150 MHz) of compounds **1**, **2**, and **5** (1 in DMSO-*d*_6_, 2 in DMSO-*d*_6_: D_2_O (1:4) and 5 in DMSO-*d*_6_:D_2_O (10:3), *δ* in ppm, *J* in Hz.).

No.	1		2		5	
	*δ*_H_ (*J*)	*δ*_C_, Type	*δ*_H_ (*J*)	*δ*_C_, Type	*δ*_H_ (*J*)	*δ*_C_, Type
2	7.36, d (2.1)	124.1, CH	7.15, s	128.9, CH	7.08, s	124.6, CH
3		110.3, C		110.8, C		109.3, C
4	7.20, d (2.2)	109.0, CH		112.9, C	6.84, d (2.1)	103.0, CH
5		146.6, C		154.8, C		150.8, C
6	6.97, dd (8.7,2.2)	111.2, CH	6.63, d (8.7)	113.6, CH	6.60, dd (8.6,2.1)	112.5, CH
7	7.22, d (8.7)	116.9, CH	7.31, d (8.1)	118.1, CH	7.15, d (8.6)	113.0, CH
8		133.4, C		133.8, C		131.7, C
9		126.8, C		125.4, C		128.3, C
10	3.00, t (7.2)	20.6, CH_2_	3.32, m	23.6, CH_2_	2.91, t (7.2)	22.6, CH_2_
11	3.27, t (7.2)	57.1, CH_2_	3.23, m	69.1, CH_2_	3.07, t (7.2)	47.8, CH_2_
13	2.82, s	42.6, CH_3_	2.98, s	54.6, CH_3_	3.30, s	50.1, CH_2_
14	2.82, s	42.6, CH_3_	2.98, s	54.6, CH_3_		169.1, C
15			2.98, s	54.6, CH_3_		
16				176.9, C		

**Table 2:** ^1^H-NMR, ^13^C-NMR (600/150 MHz) spectroscopic data for **4**, **6**, and **8** in DMSO-*d*_6_, *δ* in ppm, *J* in Hz.

No.	4		6		8	
	*δ*_H_ (*J*)	*δ*_C_, Type	*δ*_H_ (*J*)	*δ*_C_, Type	*δ*_H_ (*J*)	*δ*_C_, Type
1		61.6, C	4.19, d(8.0)	51.9, CH	4.54, s	55.8, CH
3	3.34, s	40.5, CH_2_	2.96–3.20 (m)	39.2, CH_2_	3.16–3.47, m	40.9, CH_2_
4	2.74, m	18.7, CH_2_	2.54–2.67 (m)	20.4, CH_2_	2.65–2.77, m	18.8, CH_2_
4a		103.1, C		106.4, C		103.8, C
4b		126.8, C		127.3, C		126.9, C
5	6.65, d(2.2)	102.2, CH	6.69, d(2.0)	102.2, CH	6.65, d(1.5)	101.8, CH
6		150.8, C		150.6, C		150.5, C
7	6.54, dd(8.6, 2.2)	111.5, CH	6.55, dd(8.5,2.2)	111.2, CH	6.52, dd(8.5, 1.9)	111.0, CH
8	7.15, d(8.6)	112.3, CH	7.07, d(8.6)	111.5, CH	7.19, d(8.6)	112.2, CH
8a		131.1, C		130.5, C		130.6, C
9	10.47, s		10.54, s		10.24, s	
9a		134.3, C		134.1, C		130.0, C
10		169.0, C	1.90–2.10 (m)	28.2, CH_2_		166.4, C
11	1.66, s	24.3, CH_3_	2.21–2.40 (m)	33.9, CH_2_		
12				175.3, C		
